# Glomerulonephritis with severe nephrotic syndrome induced by immune complexes composed of galactose-deficient IgA1 in primary Sjögren’s syndrome: a case report

**DOI:** 10.1186/s12882-021-02306-0

**Published:** 2021-03-25

**Authors:** Ryo Nishioka, Satoshi Hara, Hiroyuki Kawahara, Kiyoaki Ito, Ichiro Mizushima, Masayoshi Hirata, Michio Nagata, Mitsuhiro Kawano

**Affiliations:** 1 Department of Rheumatology, Kanazawa University Hospital, Kanazawa University Graduate School of Medical Science, 13-1, Takara-machi, Kanazawa, Ishikawa 920-8641 Japan; 2 Department of Nephrology, Takaoka City Hospital, 4-1, Takara-machi, Takaoka, Toyama, 933-0000 Japan; 3 Department of Kidney and Vascular Pathology, Faculty of Medicine, University of Tsukuba, 1-1-1 Tennodai, Tsukuba, Ibaraki, 305-8575 Japan

**Keywords:** Primary Sjögren’s syndrome, Renal involvement, Aberrant glycosylated IgA, Galactose-deficient IgA1, Membranous-like glomerulonephritis

## Abstract

**Background:**

Primary Sjögren’s syndrome (pSS) is an auto-immune disease characterized by sialadenitis and dacryoadenitis with lymphoplasmacytic cell infiltration. In pSS, not only sicca symptoms, but also extra-glandular involvement induced by immune abnormalities based on pSS occurs. Renal involvement is one such important life-threatening extra-glandular involvement. Although the aberrant glycosylated IgA in pSS as a product of over-activated B cells is a risk factor of renal involvement, its association has not been clarified. Here we report a case of glomerulonephritis (GN) induced by immune complexes (IC) composed of galactose-deficient IgA1 (Gd-IgA1) in a patient with pSS.

**Case presentation:**

A 48-year-old Japanese woman with pSS was admitted to our hospital because of a two-month history of nephrotic syndrome. Seven years before she had been diagnosed with pSS from keratoconjunctivitis sicca, elevation of serum anti-Ro/SSA antibody titer and lymphoplasmacytic cell infiltration around salivary ducts of the small salivary glands. Renal biopsy revealed diffuse bubbling appearance in glomerular basement membrane (GBM) with scarce mesangial proliferation. Immunofluorescence showed granular IgA, C3 and Gd-IgA1 staining of GBM. Light chain staining showed no monoclonality. Electron microscopy showed electron dense deposits mainly in the intra-membranous and paramesangial areas and slightly in the subepithelial area. Additional serum analysis confirmed elevation of Gd-IgA1 (13.5 μg/mL), which was comparable with that seen in IgA nephropathy, and qualitative enzyme-linked immunosorbent assay of IgA-containing circulating immune complex (IgA-CIC) was positive. Thus, we diagnosed GN induced by IC composed of Gd-IgA1. Furthermore, retrospectively performed immunofluorescence of the small salivary gland evaluated at the diagnosis of pSS showed positive Gd-IgA1 staining of infiltrating lymphoplasmacytic cells. Therefore, we concluded that Gd-IgA1 produced by over-activated B cells in pSS formed circulating IC and thereby induced GN. After induction therapy with high dose prednisolone and mycophenolate mofetil, the nephrotic syndrome remitted within 3 weeks, the serum Gd-IgA1 level decreased to the normal range (3.8 μg/mL), and serum IgA-CIC disappeared in the 6th month after induction therapy.

**Conclusions:**

Our findings clearly demonstrate an association between aberrant glycosylated IgA and the renal involvement seen in pSS, thereby helping to clarify the renal significance of aberrant glycosylated IgA in pSS.

## Background

Primary Sjögren’s syndrome (pSS) is an auto-immune disease characterized by sialadenitis and dacryoadenitis with lymphoplasmacytic cell infiltration. The appearance of aberrant glycosylated immunoglobulins based on B cell over-activation in pSS patients has been described, [1] but the nature of its pathogenic role and association with extra-glandular involvement have not yet been fully determined.

Renal involvement is one of the life-threatening extra-glandular manifestations and presents various forms such as tubulointerstitial nephritis, membranoproliferative glomerulonephritis (GN), mesangial proliferative GN, membranous nephropathy (MN) and rarely IgA nephropathy (IgAN) [2]. Glomerular lesions in particular are often associated with cryoglobulinemia and a worse prognosis.

We report here one pSS patient with GN induced by immune complexes (IC) composed of galactose-deficient IgA1 (Gd-IgA1) presenting nephrotic syndrome (NS). Additional investigation of Gd-IgA1 revealed both its origin in a glandular lymphoplasmacytic lesion associated with pSS and its pathogenicity as a component of circulating IC which contributed to the activity of NS. In addition, the atypical form of Gd-IgA1 deposition encountered here highlights the possibility that the Gd-IgA1 of pSS and IgAN may exhibit some clinically significant differences.

## Case presentation

A 48-year-old Japanese woman was admitted to hospital because of a two-month history of NS. Seven years before admission, she had been diagnosed with pSS from keratoconjunctivitis sicca, elevated serum anti-Ro/SSA antibody titer and lymphoplasmacytic cell infiltration around salivary ducts of the small salivary glands. At no time had she developed any extra-glandular manifestations. Although anti-nuclear antibodies were positive with a speckled pattern, no signs/symptoms satisfied the classification criteria of systemic lupus erythematous. Although a persistently elevated serum IgA level (400–500 mg/dL) was noted, she had no history of macrohematuria accompanied by upper-airway infection. In addition, there was no evidence of hypocomplementemia, cryoglobulinemia or M-protein. She had no pertinent family history, medication use, smoking or alcohol consumption habit, or occupational history.

Two months before admission, she noticed gradually appearing symmetric pedal edema. Urinalysis revealed 6.7 g/day urinary protein but no glomerular hematuria or cellular casts. On admission, her general appearance and vital signs were stable. She had no fever or malaise, and a good appetite. Her height was 152.6 cm, and weight was 45.9 kg, representing a gain of 5 kg from baseline. Physical examination showed no significant findings except for pitting edema in the bilateral lower limbs. Blood tests showed hypoalbuminemia (2.2 mg/dL) with normal kidney function (serum creatinine 0.64 mg/dL). No electrolyte imbalance was noted.

**Fig. 1:**
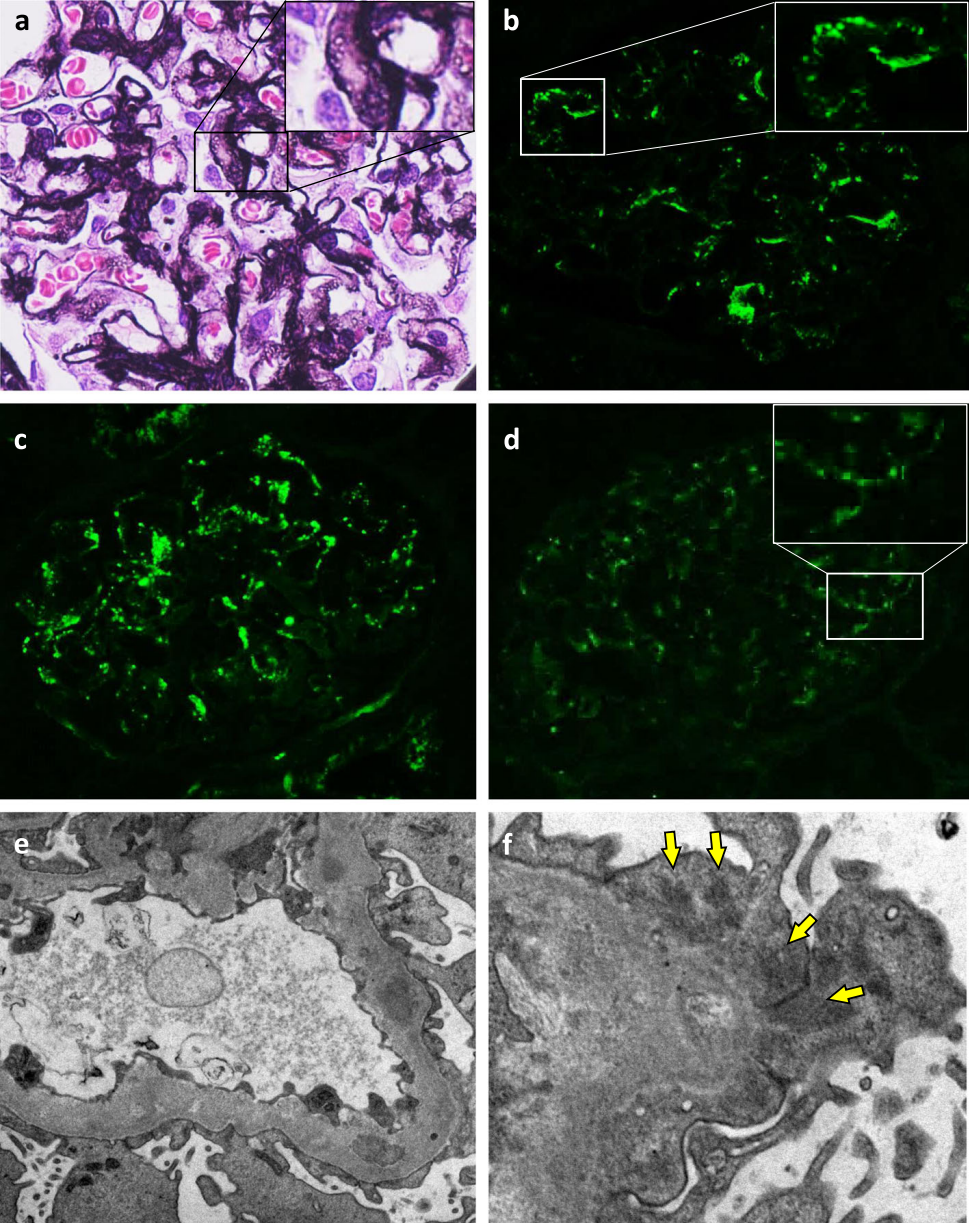
Kidney biopsy histopathology. Membranous-like glomerulonephritis with immune complex composed of galactose-deficient IgA1 (Gd-IgA1) in the patient with primary Sjögren’s syndrome. **a** Glomeruli showed diffuse bubbling appearance of glomerular basement membrane (periodic acid methenamine silver stain; original magnification, × 400). **b** Immunofluorescence (IF) for IgA showed granular deposition along glomerular capillary walls in addition to paramesangial areas (original magnification × 200). **c** IF for C3 showed the same pattern as IgA (original magnification × 200). **d** IF for Gd-IgA1 showed granular deposition along glomerular capillary walls with a pattern similar to those of IgA and C3. (original magnification × 200). For immunostaining, the primary antibody was anti-human Gd-IgA1 rat IgG monoclonal antibody from Immuno-Biological Laboratories (catalog no. 10777). **e** Ultrastructural studies showed electron dense deposits in the intra-membranous, paramesangial areas with diffuse thickening of glomerular basement membrane. Diffuse podocyte foot process effacement was also noted. (original magnification × 15000). **f** Electron dense deposits were also located in the subepithelial area (arrows). (original magnification × 25000). **g** IF for Gd-IgA1 in the small salivary gland evaluated at the diagnosis of pSS showed positive Gd-IgA1 staining of infiltrating lymphoplasmacytic cells. (original magnification × 200). **h** Negative control of IF for Gd-IgA1; the small salivary gland of a patient with pSS. (original magnification × 200)

**Fig. 2:**
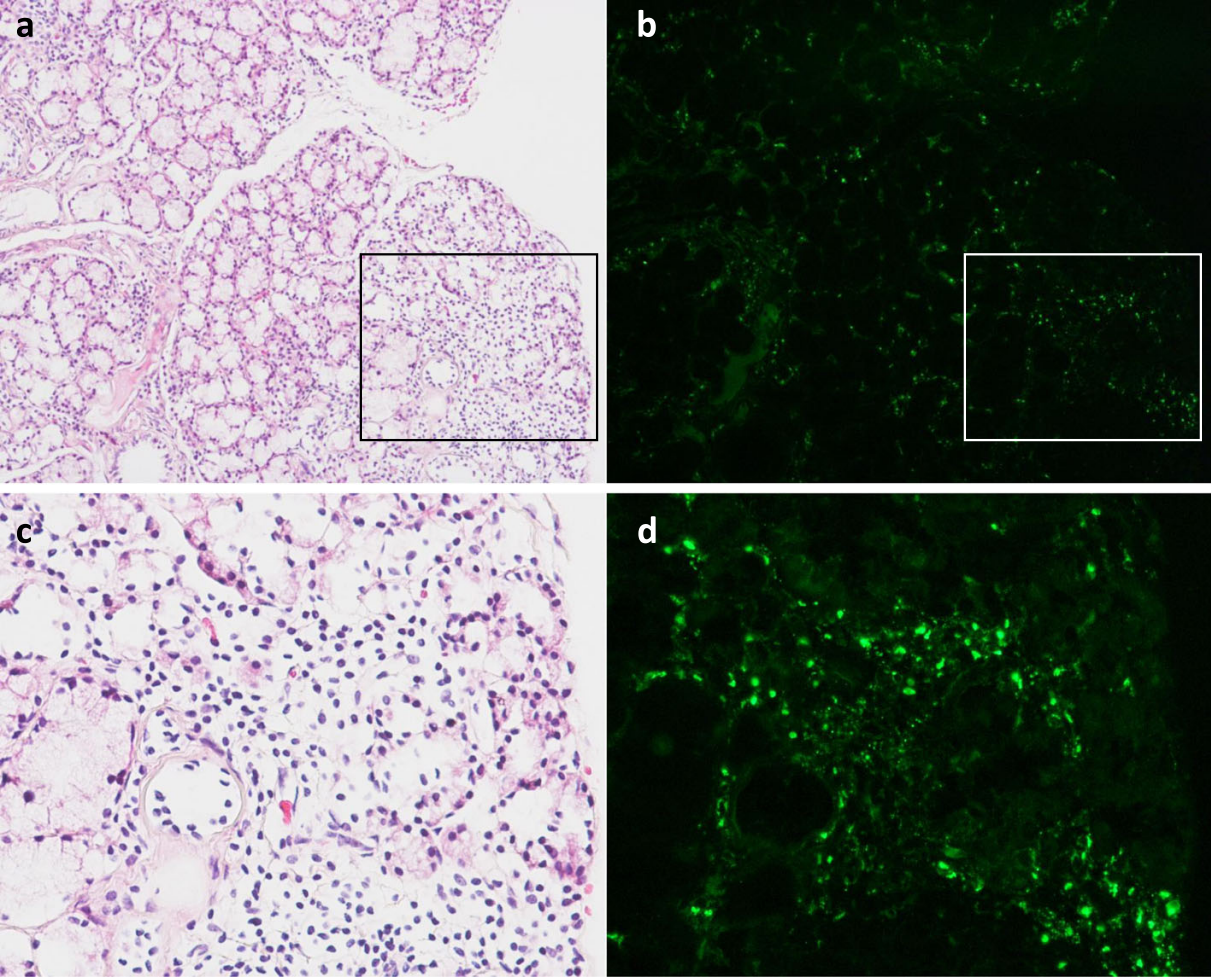
Lip biopsy histopathology. **a** Lymphoplasmacytic infiltration around salivary ducts of small salivary glands. (hematoxylin eosin stain; original magnification × 100). **b** Anti-human Gd-IgA1 staining was significantly positive in lymphoplasmacytic infiltration around salivary ducts. (original magnification × 100). **c** Image of magnified area in the square of **a**. (hematoxylin eosin stain; original magnification × 200). d Image of magnified area in the square of **b**. (original magnification × 200)

Renal biopsy showed 11 glomeruli, one of which showed global sclerosis. In the other glomeruli, diffuse bubbling appearance in the glomerular basement membrane (GBM) and slight mesangial proliferation were confirmed (Fig. 1a). There were no crescents, endocapillary proliferation, double contour of GBM, interstitial nephritis or vasculitis. Immunofluorescence (IF) revealed granular IgA and C3 deposition in GBM (Fig. 1b and c) but was negative for IgG. Light chain staining indicated no monoclonality. Anti-human Gd-IgA1 antibody (KM55) (Immuno-Biological Laboratories, Cat. No.:10777) staining was significantly positive in GBM with a granular pattern (Fig. 1d). In electron microscopy, a few electron dense deposits located in the subepithelial area, and more so in intra-membranous and paramesangial lesions accompanied diffuse GBM thickening (Fig. 1e and f). Diffuse podocyte foot process effacement was also observed. Screening for secondary MN showed no evidence of neoplasms or infection of hepatitis B virus, hepatitis C virus or human immunodeficiency virus. Additional serum analysis confirmed an elevated Gd-IgA1 level (13.5 μg/mL), comparable to that seen in IgAN, [3] and qualitative enzyme-linked immunosorbent assay (ProGen Biologics, Cat. No.:IC-003) of IgA-containing circulating immune complex (IgA-CIC) was positive. Thus, we diagnosed GN induced by IC composed of Gd-IgA1. Furthermore, retrospectively performed immunofluorescence of the small salivary gland evaluated at the diagnosis of pSS showed positive Gd-IgA1 staining of infiltrating lymphoplasmacytic cells (Fig. 2). Therefore, we concluded that Gd-IgA1 produced by over-activated B cells in pSS formed circulating IC and GN.

**Fig. 3:**
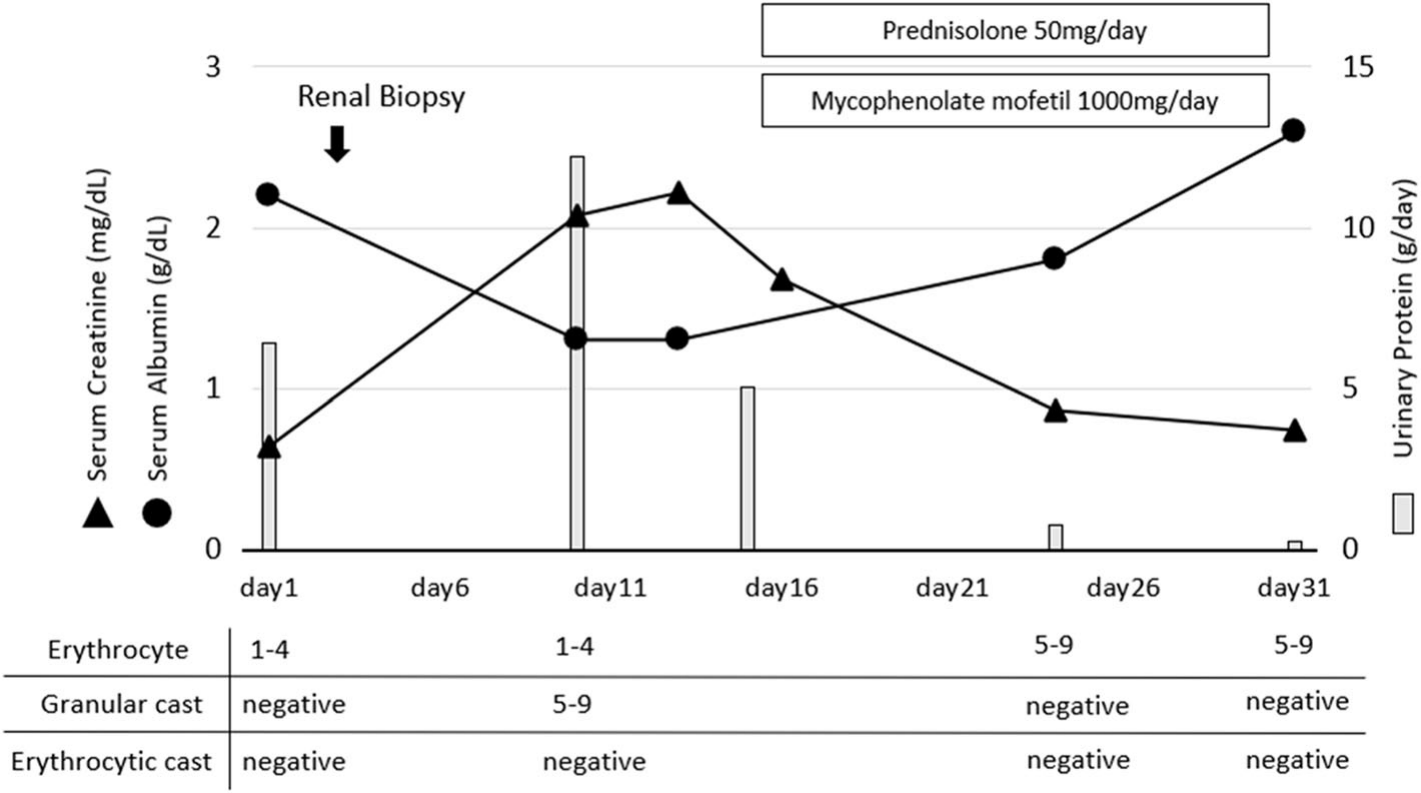
Clinical course and renal parameters during follow-up in the hospital. Serum creatinine, serum albumin, urinary protein and urinary sediment were recorded. Nephrotic syndrome deteriorated rapidly, resulting in acute kidney failure on the 10th day after admission. After induction therapy with high dose prednisolone (1 mg/kg/day) and 1 g per day of mycophenolate mofetil, the amount of urinary protein reduced dramatically and renal function recovered

NS deteriorated rapidly, with the urinary protein elevated to 12 g/day by the 10th day after admission which resulted in nephrotic crisis requiring hemodialysis for 1 week (Fig. 3). Induction therapy with high dose prednisolone (PSL) (1 mg/kg/day) and 1 g/day of mycophenolate mofetil (MMF) were administered on the 17th day, after which the amount of urinary protein reduced dramatically. NS remitted on the 24th day and she recovered baseline renal function by discharge. Five months after discharge, the remission was maintained with 1 g/day of MMF and PSL tapered to 9 mg/day. Moreover, in the 6th month after induction therapy, the serum Gd-IgA1 level decreased to the normal range (3.8 μg/mL), [3] and serum IgA-CIC disappeared.

## Discussion and conclusions

Our findings clearly demonstrate an association in pSS between aberrant glycosylated IgA and renal involvement. Although the appearance of aberrant glycosylated immunoglobulin based on B cell over-activation in pSS has been reported, [1, 4] its significance remains unclear. The present case may be of help in clarifying the clinical significance of aberrant glycosylated IgA in pSS.

The first salient point of the present case is that IC composed of Gd-IgA1 induced GN in this patient with pSS. Although the pathogenicity of aberrant glycosylated immunoglobulin in pSS has not been clarified as well as Gd-IgA1 has been in IgAN, a few past reports indicate that aberrant glycosylated IgA in pSS is correlated with elevation of serum IgA, existence of circulating IgA-CIC, and renal involvement [1, 5, 6]. The present case likewise showed a persistently elevated serum IgA and the existence of circulating IgA-CIC, in addition to Gd-IgA1 comparable with IgAN. Gd-IgA1 has been extensively investigated as a specific pathogen inducing IgAN and IgA vasculitis, [3] while some recent reports have documented that other kidney diseases also often show IC composed of Gd-IgA1 in the glomeruli such as lupus nephritis and primary MN [7, 8]. Although the frequency and pathogenicity of Gd-IgA1 in pSS are unknown, aberrant IgA in pSS has been reported to be associated with augmentation of sialylated IgA1 and IgA2, as well as reduction of galactosylated IgA1 and IgA2 [4]. In the present case at least, the detection of Gd-IgA1 in lymphoplasmacytic infiltrates implies a causal relation between Gd-IgA1 and pSS, while the rapid remission accompanying reduction of serum Gd-IgA1 level and disappearance of IgA-CIC also imply the pathogenicity of circulating IC composed of Gd-IgA1. In addition, the detection of Gd-IgA1 in glandular lymphoplasmacytic infiltration associated with pSS indicates that various lesions have the possibility to produce Gd-IgA1. In fact, a recent case report of multicentric Castleman’s disease with Gd-IgA1 dominant GN supports our contention [9]. In that report, Gd-IgA1 producing plasma cells were detected by anti-Gd-IgA1 staining of lymph nodes. Taken together, glandular Gd-IgA1 secreted by infiltrating lymphoplasmacytes could form IgA-CIC and thereby induce GN in the kidney.

The second noteworthy point is rapid resolution of NS with high dose PSL plus MMF. The treatment of GN as a renal involvement of pSS has not been established and the clinical spectrum is broad. Previously 33 therapeutic trials of 27 cases with GN in pSS has been reported (Table 1) [10–12]. Of all 27 cases in these reports, 12 cases (44.4%) finally achieved remission and 21 cases (77.8%) showed a favorable outcome (remission or amelioration). On the other hand, 2 cases progressed to end stage of renal disease despite intensive immunosuppressive treatment and one case died of lymphoma. Of all 33 therapeutic trials, PSL was most widely used (28/33, 84.8%) followed by cyclophosphamide (CY) (11/33, 33.3%). Of 12 remission cases, 7 cases (58.3%) were treated by mPSL pulse plus intravenous CY and each remaining case was treated by moderate PSL plus MMF, mPSL pulse plus azathioprine, mPSL pulse plus cyclosporine, or moderate PSL plus rituximab and rituximab solely. Because the activated B-cell lineage in pSS is supposed to form GN, such as in the present case, CY, MMF and rituximab, which selectivity suppress lymphocytic function, might be reasonable therapeutic agents. Indeed, our case demonstrated that the induction of high dose PSL and MMF rapidly decreased Gd-IgA containing IC and resolved NS.

**Table 1:** Literature review with regard to therapeutic strategy and outcome of glomerulonephritis in patients with Sjögren’s syndrome

Reference No.	Characteristics at the diagnosis of GN							Treatment	Duration until outcome	Outcome	Comments
	age	SS duration (years)	Serum Cr (mg/dL)	Urinary analysis	Histological findings	Cryoglobulin	Lymphoma				
[10]	64	11	1.4	proteinuria, hematuria	MPGN	N/A	-	mPSL pulse + IVCY	> 6 months	remission	
	50	12	0.9	proteinuria, hematuria	MSGN	N/A	-	mPSL pulse + IVCY	> 6 months	remission	
	60	8	4.9	proteinuria, hematuria	MPGN	N/A	-	mPSL pulse + IVCY	> 6 months	ESRD	
	50	18	0.8	proteinuria, hematuria	MPGN	N/A	-	mPSL pulse + IVCY	> 6 months	remission	
	66	28	1	proteinuria	MSGN	N/A	-	mPSL pulse + AZA or CsA	N/A	remission	
	44	5	1.1	proteinuria, hematuria, RBC cast	MSGN	N/A	-	moderate to high dose PSL	N/A	amelioration	very good clinical and laboratory response
	68	15	1.5	proteinuria	MSGN	N/A	-	moderate to high dose PSL	N/A	amelioration	very good clinical and laboratory response
	53	17	6.2	proteinuria, hematuria, RBC cast	GN, IN	N/A	-	mPSL pulse + IVCY	> 6 months	ESRD	
	74	27	1.6	proteinuria	MPGN, IN	N/A	-	no treatment	13 years	death	developed lymphoma
	53	10	1.1	proteinuria, hematuria	MSGN	N/A	-	RTX	N/A	remission	
	75	15	0.9	proteinuria, hematuria	MPGN	N/A	-	mPSL pulse + IVCY	> 6 months	remission	
	38	11	0.7	proteinuria, hematuria	MPGN	N/A	-	mPSL pulse + IVCY	< 2 months	remission	
	42	7	1	proteinuria, hematuria	MPGN	N/A	-	moderate to high dose PSL	<1 years	amelioration	very good clinical and laboratory response
	39	10	1.5	proteinuria	MPGN	N/A	-	mPSL pulse + IVCY	< 2 months	remission	
	25	4	0.8	proteinuria	MPGN	N/A	-	moderate to high dose PSL	N/A	amelioration	very good clinical and laboratory response
	42	12	1	proteinuria, hematuria	MPGN	N/A	+	R-CHOP	<1 years	stable	
	75	31	0.8	hematuria	MSGN, IN	N/A	-	no treatment	23 years	N/A	
	55	14	1.2	proteinuria, hematuria	MSGN, IN	N/A	+	chemotherapy without RTX	N/A	amelioration	
	43	15	1.5	normal finding	proliferative GN, IN	N/A	-	mPSL pulse + IVCY	<1 years	remission	
	59	8	0.9	hematuria	MN	N/A	-	moderate to high dose PSL	N/A	amelioration	
	65	8	0.9	proteinuria	MN	N/A	-	mPSL pulse + AZA or CsA	N/A	remission	
[11]	N/A	7	2	proteinuria, hematuria	MPGN, IN	+	-	① moderate dose PSL + HCQ	① 24 months	① detelioration	
								② moderate dose PSL + RTX	② 5 months	② amelioration	
	N/A	0	1.6	proteinuria, hematuria	Fibrially GN	-	-	① high dose PSL	① 2.5 months	① detelioration	
								② moderate dose PSL + RTX	② 5 months	② detelioration	
	N/A	20	0.8	normal finding	MPGN	+	-	① moderate dose PSL + MMF	① 10 months	① stable	complication of vasculatic ulcers
								② high dose PSL + oral CY	② 3 months	② stable	
								③ moderate dose PSL + MMF	③ 3 months	③ remission	
	N/A	11	0.9	proteinuria, hematuria	MPGN	+	-	① low dose PSL + HCQ + colchicine	① 4 years	① stable	
								② high dose PSL + MMF	② 12.5 months	② detelioration	
								③ moderate dose PSL + RTX	③ 6 months	③ remission	
[12]	58	N/A	3.5	proteinuria	MPGN, IN	+	-	PSL	<1 months	amelioration	
	47	N/A	1.4	proteinuria, hematuria	MPGN, IN	+	-	PSL + HCQ, RTX added 12 years after	12 years	amelioration	developed lymphoma 12 years after diagnosis of GN

*N/A* no available, *GN* glomerulonephritis, *MP* membranoproliferative, *MS* mesangial, *IN* interstitial nephritis, *SS* Sjögren’s syndrome, *Cr* creatinine, *ESRD* end stage renal disease, *mPSL* methyl-prednisolone, *IVCY* intravenous cyclophosphamide, *MMF* mycophenolate mofetil, *RTX* rituximab, *AZA* azathioprine, *CS* cyclosporine, *HCQ* hydroxychloroquine, *RCHOP* rituximab, cyclophosphamide, doxorubicin hydrochloride, vincristine and prednisolone

Finally, the present case does not represent merely a combination of IgAN and MN. Patients with coexisting IgAN and MN have been reported as a clinically distinctive group [13]. They tend to have NS, serum Gd-IgA1 level comparable with IgAN, and a lower frequency of gross hematuria than IgAN. Although these clinical features are similar to those of the present case, our case is quite different because of the scarce mesangial proliferation and granular IgA deposition along the GBM seen. In addition, our case is also different from two previous ones of MN with solitary polyclonal IgA deposits [14, 15]. Although they are different from our case in terms of lacking C3 deposition and subepithelial-dominant electron dense deposition, it should be noted that polyclonal IgA can pass through GBM and deposit at subepithelial sites.

In conclusion, aberrant glycosylated IgA as a product of immune abnormality in pSS has the potential to induce IC-mediated GN causing severe NS. More reports on similar cases will be required to validate this conclusion.

## Data Availability

All the data relevant to this report are included in the manuscript.

## Abbreviations

pSS: Primary Sjögren’s syndrome
Gd-IgA1: Galactose-deficient IgA1
GN: Glomerulonephritis
IC: Immune complex
GBM: Glomerular basement membrane
IgA-CIC: IgA-containing immune complex
MN: Membranous nephropathy
IgAN: IgA nephropathy
NS: Nephrotic syndrome
PSL: Prednisolone
MMF: Mycophenolate mofetil
CY: Cyclophosphamide
